# Aligning Preprofessional Student Experiences in Integrative Group Medical Visits with Integrative Medicine Core Competencies

**DOI:** 10.1089/imr.2022.0052

**Published:** 2022-10-03

**Authors:** Malik K. Tiedt, Bethany M. Kavalakatt, Aisha Chilcoat, Jessica L. Barnhill, Isabel J. Roth

**Affiliations:** Program on Integrative Medicine, Department of Physical Medicine and Rehabilitation, University of North Carolina at Chapel Hill, Chapel Hill, NC, USA

## Introduction

Wide utilization of complementary and integrative health care (CIH) approaches by the American public has warranted an increase in the presence and quality of CIH-focused education.^1^ Among U.S. medical schools with integrative medicine training programs, passive instruction rather than active involvement in clinical delivery is the norm.^2^ Future health professionals may benefit from innovative educational models framed around the core competencies for integrative medicine established by the Academic Consortium for Integrative Medicine and Health (ACIMH).^3,4^

One model that incorporates students into the delivery of CIH services is the integrative group medical visit (IGMV): a program offering peer support, education about nutrition, acupressure, mindfulness, yoga, sleep hygiene, and more.^5,6^ IGMVs increase access to CIH services, decrease emergency department utilization, and decrease pain medication usage in populations with chronic pain.^7,8^ Involvement in these programs present students with an opportunity to learn about CIH modalities and their delivery in underserved populations.

Prior articles have discussed IGMVs as a component of preventive medicine residency training, but little has been said about *how* trainee experiences align with ACIMH's core competencies.^9^ This commentary articulates, from students' perspectives, how the IGMV (1) teaches the ACIMH's integrative medicine core competencies and (2) offers a unique approach to actively involve future providers in CIH delivery.

## Student Responsibilities and Involvement

Viewpoints in this article originate from an undergraduate student (M.K.T.), current medical student (B.M.K.), postdoctoral fellow (A.C.), and two faculty members (J.L.B. and I.J.R.) at an academic program on integrative medicine.^10^ Students were involved in two virtual IGMVs: one for patients with chronic pain and another adapted to populations experiencing long-COVID. These IGMVs drew from a previously developed curriculum,^7^ and were embedded in an outpatient integrative medicine clinic with a high burden of patients with chronic pain. Implementation mapping guided efforts to IGMV delivery,^11^ and the program was found to be feasible and acceptable to patients and providers.^12–14^

Students interviewed clinical stakeholders, assisted in the development of an implementation protocol, consented participants, conducted pre- and postintervention interviews, and analyzed data. This allowed students to submit abstracts, present at conferences, and assist in article preparation. Students also assisted in adapting the curriculum for telehealth delivery, guided patients through technology issues, constructed a website for IGMV materials, and facilitated discussions on topics of interest.

## Mapping Student Experiences to Integrative Medicine Core Competencies

Conventional clinical training may include ACHIM's integrative medicine core competencies (patient care, medical knowledge, practice-based learning, communication, professionalism, and systems-based practice) at varying degrees; however, clinical practice centered on CIH prioritizes these principles.^15^ Providers must consider how modalities are presented and received by patients, the context in which this occurs, and the way these services influence patient–provider interactions. Participating in an IGMV is an innovative method of clinical education conducive toward training students in CIH delivery. In this study, students discuss how the model expanded their clinical skills and capacities to provide higher quality care.

### Core competency 1: Patient care

The IGMV taught patient-directed care by prioritizing compassion as an essential component of group-based clinical services. Students witnessed provider responsibilities shift within a group setting as facilitators created a social space where patients could bond over adapting new health behaviors or coping with their symptoms. Facilitators were also observed employing active listening skills to help patients identify their individual goals: a skill that can better align treatment plans with a patient's cultural and health belief systems.

Students recognized the challenges patients experience while receiving care and living with chronic conditions as well. For instance, they learned about feelings of frustration and isolation in the context of chronic pain, and how provider uncertainty contributes to feelings of helplessness among those experiencing long-COVID. In addition, students developed capacity to identify which patient personalities are well suited for group-based services: extroverted patients generally embraced group discussions, whereas more reserved individuals may prefer individual meetings with their providers.

### Core competency 2: Medical knowledge

Students learned about the biomedical, health, and social sciences relevant to the field of integrative medicine in the IGMV model by identifying providers as informational liaisons who translate CIH research into practice. Facilitators were ultimately responsible for engaging with literature and clearly communicating its findings, particularly for long-COVID patients where little was known about symptom etiology and treatment. Students were placed within this role when they evaluated literature and drew on prior trainings (i.e., Registered Yoga Teacher training) to develop yoga activities or nutrition discussions under the guidance of a mentor. This exposure to CIH research gave students an opportunity to practice translating scientific findings into clinical settings. In addition, involvement in IGMV discussions led by guest practitioners (i.e., Registered Dietitians) gave students an opportunity to learn about modalities not explicitly included in the IGMV curriculum.

### Core competency 3: Practice-based learning and improvement

The IGMV educated students about the third core competency by exposing them to patient feedback and inspiring involvement in CIH research. Facilitators of the adapted groups for long-COVID symptoms elicited and responded to patient feedback through the use of weekly intake surveys. Witnessing how providers used this information to adjust the curriculum and structure of the IGMV exemplified how patient concerns are prioritized in the delivery of group-based CIH services.

Students directly participated in practice-based learning by developing independent research questions or projects related to IGMV. Mentors led students through this process and elicited their assistance in developing grant proposals, leading conference presentations, and drafting articles. This advanced how students engaged with CIH research and taught them valuable clinical research skills.

### Core competency 4: Interpersonal and communication skills

The IGMV taught students how to foster effective respectful relationships with both patients and other practitioners involved in group facilitation. In comparison with conventional shadowing experiences, the patient–provider hierarchy in IGMVs was observed to be flattened, largely due to the ground rules established in partnership with patients during the first group meeting (e.g., not interrupting others, asking others for clarification, and speaking from your own experience). The multidisciplinary team of providers involved within IGMV implementation (doctor of public health, naturopathic doctor, medical doctor) also taught students about how healthy interprofessional collaboration between referring providers and administrators is needed for the successful delivery of CIH.

### Core competency 5: Professionalism

Professionalism was taught within the IGMV as students retained clinical responsibilities, observed how providers established inclusivity, and developed capacity to recognize signs of burnout. Students played a critical role in ensuring successful IGMV delivery as they helped patients navigate technology, sent out weekly emails with meeting information, and made group resources available online. In terms of inclusivity, students learned that safe spaces are not simply assumed: it is a responsibility of the provider to explicitly establish a comfortable environment. Students also developed professionalism by learning the fundamentals of practicing mindfulness and self-care: techniques they could utilize to prevent burnout and enhance the quality of care.

### Core competency 6: Systems-based practice

The last competency is targeted toward understanding CIH's role within the broader health care system. IGMV involvement helped students understand this ideal based on the type of patients referred to the groups as well as some of the administrative barriers to implementation. Students learned about the conversations that needed to happen as part of CIH implementation: meeting with administrators about billing concerns, establishing shared values with clinicians, and engaging stakeholders to establish a robust referral network. Students also grasped how different patients received CIH services: some were entirely invested, whereas others simply attended the groups due to a lack of other options. Either way, students learned about the variety of patients referred to IGMV for chronic pain or long-COVID as well as how CIH approaches can support these populations.

## Final Remarks

Students felt that IGMV programs educated them about the ACIHM's established core competencies, and bolstered their capacity as future providers beyond what would be learned in conventional clinical shadowing experiences. Because the IGMV model is centered around increasing the accessibility and affordability of CIH services to conventionally underserved populations,^5^ students were challenged to consider how patient diversity and health equity could be incorporated into their future practice.

Although the IGMVs discussed target chronic pain and long-COVID, this curriculum has potential to serve populations with diabetes, cancer, mental health needs, and more.^6^ Involving various future providers in this model at multiple levels of education—undergraduate, graduate, medical, and residency—could teach CIH competencies across specialties and may improve multidisciplinary collaboration as a wide range of professionals (physicians, advanced practice nurses, physician assistants, pharmacists, dentists, and naturopathic doctors) have completed integrative medicine fellowships.^16^

Student reflections presented within this commentary were shaped by mentors with qualifications and years of experience in CIH. The combination of this professional guidance and interaction with stakeholders who supported IGMV implementation as part of a growing integrative medicine program may not be shared across contexts. Further evaluation is needed to inform how IGMVs meet a variety of patients' needs and support CIH education in diverse clinical settings.

## Abbreviations Used

ACIMH: Academic Consortium for Integrative Medicine and Health
CIH: complementary and integrative health care
IGMV: integrative group medical visit
